# Combination of LC3 shRNA Plasmid Transfection and Genistein Treatment Inhibited Autophagy and Increased Apoptosis in Malignant Neuroblastoma in Cell Culture and Animal Models

**DOI:** 10.1371/journal.pone.0078958

**Published:** 2013-10-18

**Authors:** Nishant Mohan, Mrinmay Chakrabarti, Naren L. Banik, Swapan K. Ray

**Affiliations:** 1 Department of Pathology, Microbiology, and Immunology, University of South Carolina School of Medicine, Columbia, South Carolina, United States of America; 2 Department of Neurosciences, Medical University of South Carolina, Charleston, South Carolina, United States of America; University of Chicago, United States of America

## Abstract

Malignant neuroblastoma is an extracranial solid tumor that usually occurs in children. Autophagy, which is a survival mechanism in many solid tumors including malignant neuroblastoma, deters the efficacy of conventional chemotherapeutic agents. To mimic starvation, we used 200 nM rapamycin that induced autophagy in human malignant neuroblastoma SK-N-BE2 and IMR-32 cells in cell culture and animal models. Combination of microtubule associated protein light chain 3 short hairpin RNA (LC3 shRNA) plasmid transfection and genistein (GST) treatment was tested for inhibiting rapamycin-induced autophagy and promoting apoptosis. The best synergistic efficacy caused the highest decrease in cell viability due to combination of 50 nM LC3 shRNA plasmid transfection and 25 µM GST treatment in rapamycin-treated SK-N-BE2 cells while combination of 100 nM LC3 shRNA plasmid transfection and 25 µM GST treatment in rapamycin-treated IMR-32 cells. Quantitation of acidic vesicular organelles confirmed that combination of LC3 shRNA plasmid transfection and GST treatment prevented rapamycin-induced autophagy due to down regulation of autophagy promoting marker molecules (LC3 II, Beclin 1, TLR-4, and Myd88) and upregulation of autophagy inhibiting marker molecules (p62 and mTOR) in both cell lines. Apoptosis assays showed that combination therapy most effectively activated mitochondrial pathway of apoptosis in human malignant neuroblastoma in cell culture and animal models. Collectively, our current combination of LC3 shRNA plasmid transfection and GST treatment could serve as a promising therapeutic strategy for inhibiting autophagy and increasing apoptosis in human malignant neuroblastoma in cell culture and animal models.

## Introduction

 Malignant neuroblastoma is the most frequently diagnosed and highly aggressive extracranial solid tumor that mainly occurs in children.  It most commonly arises from adrenal medulla or abdominal sympathetic ganglia and exhibits very complex biological and clinical heterogeneity [1,2]. While very young children have significant probability of spontaneous regression or complete remission with conventional treatment, substantial number of older patients can show progressive malignancy despite multimodal intensive therapy. Initiation and progression of malignant neuroblastoma are attributed to a variety of genetic aberrations including deletion of chromosome 1p and 11q, addition of chromosome 17q, and amplification of N-Myc oncogene [3,4]. The rising incidence and relapse of malignant neuroblastoma and its poor prognosis coupled with modest survival rate of patients are compelling reasons to identify innovative and novel therapeutic strategies for proper management of this pediatric malignancy. 

 Autophagy, which is an evolutionary conserved catabolic process that plays critical role in homeostatic removal with degradation and recycling of damaged and mis-folded proteins and organelles, affects various physiological and pathological processes [5,6]. The role of autophagy in various cancers is highly complex and not well understood yet. Currently, it appears that autophagy is an important process in solid tumors to utilize nutrients and provide building blocks for growth of tumor cells during adverse circumstances such as oxygen depletion and starvation and thus autophagy contributes to overall survival of tumor cells [7,8]. Inhibition of autophagy by combination of genetic approach and pharmacological intervention is being explored for controlling growth of solid tumors in cell culture and animal models. Emerging data suggest that autophagy plays a dual role in cell survival as well as in cell demise; however, crosstalk and interplay between autophagy and apoptosis appear to be complex and also controversial [9]. Autophagic cells form double membrane bound vesicles called autophagosomes, which engulf degrading cytoplasm and cytoplasmic organelles, thus function as protective players to allow recycling of cellular components so as to bolster survival of other tumor cells. Mammalian target of rapamycin (mTOR) signaling plays an essential role in negative regulation of autophagy by influencing the formation of autophagosomes at early stages [10]. Rapamycin treatment mimics starvation, thus rapamycin is a widely known autophagy inducer and specific inhibitor of mTOR signaling, and rapamycin blocks the functions of mTOR by inhibiting phosphorylation of downstream signaling molecules to induce autophagy [11,12]. 

 Microtubule associated protein light chain 3 (LC3), which is a mammalian counterpart of yeast Atg8, is a highly sensitive molecular marker of autophagosome and thus LC3 is extensively used as an indicator to monitor autophagic activity [13,14]. Human isoform of LC3 undergoes post-translational modification during autophagy and generates cytosolic LC3 I form by cleaving LC3 at carboxy terminus. Subsequently, LC3 I can undergo lipidation for conversion to LC3 II form, which then gets associated with autophagosomal membranes. 

Small interfering RNA (siRNA) technology is an extremely popular and powerful tool that is used for silencing expression of specific gene in the cells, preferably by delivery of a plasmid construct containing the sequence of short hairpin RNA (shRNA). The hairpin structure in shRNA is cleaved by the RNA endonuclease Dicer into short (~ 22 nucleotides) double stranded siRNA containing the passenger strand and the guide strand, the passenger strand is degraded while the guide strand is incorporated into the RNA-induced silencing complex (RISC), and then RISC uses the guide strand as a template to find the complementary mRNA of a specific gene for its cleavage, resulting in inhibition of its translation [15].

 Genistein (GST), which is a specific inhibitor of protein tyrosine kinase, has recently garnered wide spread attention all over the world because of its emerging roles in reducing cancer risks. GST is a soy derived isoflavone with heterocyclic diphenolic structure and it is a potent inhibitor of cell proliferation, oncogenesis, and clonogenesis without causing cytotoxicity to normal tissue [16-18]. GST induces apoptosis and thus inhibits growth of malignant tumor cells in a variety of organs including prostate, lung, ovary, breast, and colon [17-19]. It also possesses anti-angiogenic and anti-metastatic properties and therefore it is considered to be an attractive anti-tumor agent. We have previously shown the anti-tumor properties of GST alone and also in combination with other therapeutic agents in human malignant neuroblastoma cells both in vitro and in vivo, where GST has induced cell death via activation of the receptor and mitochondrial pathways of apoptosis [19-21]. 

 In the current investigation, we used LC3 shRNA plasmid transfection for inhibition of expression of LC3 mRNA leading to low levels of both LC3 I and LC3 II in human malignant neuroblastoma SN-N-BE2 and IMR-32 cell lines. The aim of this investigation was to first induce autophagy in these two genetically diverse human malignant neuroblastoma cell lines by using the potent autophagy inducer rapamycin and then examine whether the combination of LC3 shRNA plasmid transfection and GST treatment could inhibit autophagy and increase apoptosis. Our current study clearly showed the molecular mechanisms of a crosstalk between autophagy and apoptosis and indicated development of an important therapeutic strategy for complete inhibition of autophagy and huge increase in apoptosis for controlling the growth of different human malignant neuroblastoma cells in culture and animal models. 

## Materials and Methods

### Human malignant neuroblastoma cell lines and culture conditions

 Human malignant neuroblastoma SK-N-BE2 and IMR-32 cell lines were purchased from the American Type Culture Collection (ATCC, Manassas, VA, USA). The rationale behind using two different cell lines was based on the presence of different status of the tumor suppressor p53, being mutant in SK-N-BE2 cell line and wild-type in IMR-32 cell line. SK-N-BE2 cell line was propagated in RPMI 1640 (Mediatech, Manassas, VA, USA) while IMR-32 cell line was propagated in DMEM (Mediatech, Manassas, VA, USA) and both growth media were supplemented with 10% fetal bovine serum (Atlanta Biologicals, Lawrenceville, GA, USA) and 1% penicillin and 1% streptomycin (GIBCO, Grand Island, NY, USA) in a fully humidified incubator containing 5% CO_2_ and 37°C. GST (Sigma Chemical, St. Louis, MO, USA), wortmannin (WM, Tocris Bioscience, Bristol, UK), and 3-methyl adenine (3MA, Sigma Chemical, St. Louis, MO, USA) were dissolved in dimethyl sulfoxide (DMSO) to make stock solutions and aliquots were stored at -20°C until ready to use. Concentration of DMSO in all experiments was maintained at less than 0.01% that did not affect cell growth or death.

### Acridine orange (AO) staining to detect acidic vesicular organelles (AVO) in autophagic cells

 AO staining was performed to detect and quantify AVO, a highly characteristic feature of autophagic cells. Briefly, cells after treatment with 200 nM rapamycin (for 6, 12, or 24 h) were incubated with AO (1 µg/ml) for 15 min for staining. In some experiments, rapamycin-treated cells were used as treated control (CTL) and then transfected with LC3 shRNA plasmid or/and treated with GST for another 24 h followed by incubation with AO (1 µg/ml) for 15 min for staining. Slides containing the AO stained cells were then examined under the fluorescence microscope for detection of AVO in autophagic cells. We also performed flow cytometric analysis after AO staining of the cells. In flow cytometric analysis of the AO stained cells, cytoplasm and nucleolus in non-autophagic cells showed green fluorescence (500-550 nm, FL-1 channel) whereas AVO in autophagic cells (quadrant A1) showed bright red fluorescence (650 nm, FL-3 channel). Intensity of red fluorescence is proportional to the number of AVO in autophagic cells. Therefore, AVO in autophagic cells can be quantified on the basis of the intensity of red fluorescence. 

### Transfection of cells with LC3 shRNA plasmid

 Both SK-N-BE2 and IMR-32 cell lines were allowed to grow till 90% confluency and then transfected with LC3 shRNA plasmid (Santa Cruz Biotechnology, Santa Cruz, CA, USA). The human expression vector LC3 shRNA plasmid (sc-43390-SH) consists of a pool of 3 target-specific lentiviral vector plasmids (sc-43390-SHA, sc-43390-SHB, and sc-43390-SHC), each of which encodes small (19-25 nucleotides plus hairpin) shRNA molecules designed for most efficient knockdown of expression of LC3 gene in human cells. The shRNA plasmid sc-43390-SHA was used for generation of the siRNA duplex (sc-43390A) with sense sequence: 5’-GGU UUG UUC UCU AGA UAG Utt-3’ and antisense sequence: 5’-ACU AUC UAG AGA ACA AAC Ctt-3’ in the cells. The shRNA plasmid sc-43390-SHB was employed for biosynthesis of the siRNA duplex (sc-43390B) with sense sequence: 5’-CGU ACG CUC UUU ACA GAU Att-3’ and antisense sequence: 5’-UAU CUG UAA AGA GCG UAC Gtt-3’ in the cells. The shRNA plasmid sc-43390-SHC was used for production of the siRNA duplex (sc-43390C) with sense sequence: 5’-CUC GUU UAG ACU GUA UAC Att-3’ and antisense sequence: 5’-UGU AUA CAG UCU AAA CGA Gtt-3’ in the cells. Transfection was performed using Lipofectamine 2000 reagent (Invitrogen, Grand Island, NY, USA) following the manufacturer’s instruction. Lipofectamine 2000 is a widely used transfection reagent because of its high transfection efficiency and its ability to form complex with plasmid that can be directly added to the cells in a culture medium. Briefly, cells were plated in a 24-well format in 500 µl of growth medium without addition of antibiotics and allowed to grow till 90% confluency. Both plasmid DNA and Lipofectamine 2000 reagent were diluted in 50 µl of serum-free Opti-MEM (Invitrogen, Grand Island, NY, USA) medium separately and incubated for 5 min. After incubation, plasmid DNA and Lipofectamine 2000 reagent were mixed gently and added to each well containing cells and medium. Cells were incubated at 37°C for 24 h in an incubator containing 5% CO_2_ and full humidity. The CTL shRNA (Santa Cruz Biotechnology, Santa Cruz, CA, USA) consisting of the scrambled sequence, which would not lead to specific degradation of any known cellular mRNA molecules, was employed as a negative CTL in the experiments.

### The 3-(4,5-dimethylthiazolyl-2)-2,5-diphenyltetrazolium bromide (MTT) assay to determine residual cell viability

 The MTT assay was performed to determine residual cell viability after transfection of cells with LC3 shRNA plasmid or/and treatment with GST. Both SK-N-BE2 and IMR-32 cell lines were either transfected with LC3 shRNA plasmid (10, 50, and 100 nM) or treated with GST (25, 50, and 100 µM) or combination of both agents for 24 h. Cells were then exposed to the MTT reagent (10 µl/well) for 3 h at 37°C followed by addition of isopropanol (100 µl) to dissolve the formazan crystals. The optical density (OD) was recorded at 570 nm in a microplate reader (BioTek, Winooski, VT, USA). Cell viability data obtained from the MTT assay were then subjected to analysis by Compusyn software (ComboSyn, Paramus, NJ, USA) to determine combination index (CI) values. Conventionally, CI > 1 indicates antagonism, CI = 1 indicates additive effect, and CI < 1 indicates synergism of the agents in combination.

### In situ Wright staining and Annexin V-fluorescein isothiocyanate (FITC)/propidium iodide (PI) binding assay to assess apoptosis

 Both SK-N-BE2 and IMR-32 cell lines after the plasmid transfection or/and GST treatment were centrifuged and then washed twice with phosphate-buffered saline (PBS), pH 7.4. Cells were fixed in 95% (v/v) ethanol, air-dried, and then subjected to in situ Wright staining using a kit (Fisher Scientific, Kalamazoo, MI, USA) according to manufacturer’s instruction. The morphology of the cells was examined under the light microscope. The morphological features of apoptotic cells included several visible characteristics such as cell shrinkage, chromatin condensation, DNA fragmentation, and membrane bound apoptotic bodies. About 400 cells were counted from four randomly selected fields and the percentage of apoptotic cells was calculated from three separate sets of experiments. For quantitative estimation of apoptosis, Annexin V-FITC/PI binding assay followed by flow cytometry was also performed, as we recently described [20], to detect an early molecular event of apoptosis that occurred after single therapy or combination therapy. In flow cytometric analysis, cells that were Annexin V-FITC negative and PI positive were considered as mechanically injured (quadrant A1), cells that were both Annexin V-FITC and PI positive (quadrant A2) were considered as late necrotic, cells that were both Annexin V-FITC and PI negative (quadrant A3) were considered as normal, and cells that were Annexin V-FITC positive and PI negative were considered as early apoptotic (quadrant A4). Flow cytometry detected the Annexin V-FITC positive cells that experienced externalization of membrane phospholipid, an early biochemical feature of apoptosis. The Annexin V-FITC stained apoptotic cells were analyzed for statistical significance.

### Development and treatment of neuroblastoma xenografts in nude mice

 Six-week old athymic nude mice were purchased (Charles Rivers Laboratories, Wilmington, MA, USA) for our in vivo studies following the procedures, as we reported recently [20]. Our all animal studies were conducted in strict compliance with the recommendations in the ‘Guide for the Care and Use of Laboratory Animals’ of the National Institutes of Health and also with approval from the Institutional Animal Care and Use Committee (IACUC) of the University of South Carolina (Columbia, SC, USA). We developed the xenografts by implanting human malignant neuroblastoma SK-N-BE2 or IMR-32 cells (6×10^6^) in 100 µl of (1:1) mixture of 1×RPMI and Matrigel (BD Biosciences, San Jose, CA, USA) subcutaneously at the flank region of nude mice under isofluorane anesthetic condition, as we described previously [20]. After 6-8 days, palpable tumors were developed in nude mice. We used DMSO to dissolve rapamycin (25 mg/ml), which was further diluted to 1:200 (v/v) in PBS before intraperitoneal injection. After 3 weeks of xenograft developments, animals were daily pre-treated with rapamycin (2 mg/kg body weight/day) for 7 days. After 7 days of rapamycin pre-treatment, animals were randomly divided into four groups: CTL shRNA plasmid transfection, LC3 shRNA plasmid transfection, GST treatment, and LC3 shRNA plasmid transfection + GST treatment. Each animal in CTL shRNA plasmid transfection group and LC3 shRNA plasmid transfection group was daily injected with 50 µg of the CTL shRNA plasmid and 50 µg of the LC3 shRNA plasmid, respectively. The injections were performed using a Hamilton syringe with the help of a stereotaxic apparatus at tumor site. For delivery of shRNA plasmid into the mice, we used Invivofectamine 2.0 reagent (Invitrogen, Grand Island, NY, USA) that was animal-origin-free lipid based in vivo transfection reagent designed for systemic siRNA delivery with high in vivo transfection efficiency in mouse tissues. The GST treatment group received GST (2 mg/kg body weight/day) and the combination therapy group received both agents. In the combination therapy group, animals were injected with LC3 shRNA plasmid followed by GST treatment at an interval of 10 min. After all transfections and treatments for 15 days, animals were subjected to anesthesia with isoflurane and sacrificed to excise the xenografts. Tumor measurement was performed by using an external caliper and tumor volume was determined with the formula: 4π/3 x (length/2) x (width/2)^2^. We determined volume of each tumor to assess its regression due to a monotherapy or combination therapy.

### Histopathological examination of neuroblastoma xenografts

 Neuroblastoma xenografts from each treatment group were cut into two halves and one half was frozen immediately in liquid nitrogen and stored at -80°C for future use. The other half of the xenograft was frozen in Optima Cutting temperature (OCT) media (Fisher Scientific, Suwanee, GA, USA) for cutting 5 µm sections of the tumor in OCT with a cryostat (Leica, Deerfield, IL, USA). These sections were then subjected to hematoxylin and eosin (H&E) staining for examination of changes in histopathology in the tumors after the treatments, as we described recently [20]. 

### Protein extraction for sodium dodecyl sulfate-polyacrylamide gel electrophoresis (SDS-PAGE) and Western blotting

 We performed SDS-PAGE and Western blotting to assess the levels of expression of various signaling proteins, as we previously described [19,20]. Cells or tumors from all treatment groups were homogenized in lysis buffer to make lysates for extraction of cytosolic proteins. To analyze the cytosolic cytochrome c that was released from mitochondria, lysates from all treatment groups were centrifuged at 12,000g in cold (4°C) for 3 min to obtain the supernatant (cytosolic fraction without mitochondria) and the pellet (mitochondrial fraction). Cytosolic and mitochondrial protein samples were quantitated spectrophotometrically and denatured in boiling water for 5 min. Protein concentrations were determined after staining the samples with Coomassie Plus protein reagent (Pierce Biotechnology, Rockford, IL, USA) using the modified Bradford method. Proteins were resolved by SDS-PAGE and electroblotted to transfer the resolved proteins to the polyvinylidene fluoride (PVDF) membranes (Millipore, Billerica, MA, USA). After blocking in 5% non-fat milk, PVDF membranes or Western blots containing proteins were subsequently probed with specific primary IgG antibodies. To monitor the levels of cytochrome c in both mitochondrial and cytosolic fractions, the Western blots were probed with primary IgG antibody against cytochrome c. For detection of primary IgG antibodies, we used horseradish peroxidase (HRP) conjugated secondary IgG antibodies (1:2000) followed by HRP-Immunostar chemiluminescence reagent (Bio-Rad Laboratories, Hercules, CA, USA). For development of autoradiogrms, Western blots were exposed to X-OMAT AR films (Eastman Kodak, Rochester, NY, USA). The autoradiograms were scanned on an EPSON Scanner using Photoshop software (Adobe Systems, Seattle, WA, USA) to examine the levels of expression of speciﬁc proteins. The expression of cytochrome c oxidase subunit 4 (COX-4, a mitochondrial protein) was used as a loading control on Western blots with mitochondrial or cytosolic fraction. Expression of β-actin (a cytosolic protein) was used as a loading control on Western blots with cytosolic proteins.

### Statistical analysis

 Results from some experiments were analyzed using Minitab® 16 statistical software (Minitab, State College, PA, USA). Data were expressed as mean ± standard error of mean (SEM) of separate experiments (*n*≥3) after comparing with one-way analysis of variance (ANOVA) followed by the Fisher’s post-hoc test. Difference between a control (CTL) group and a treatment group or between two treatment groups was considered signiﬁcant at *P*<0.05. 

## Results

### Rapamycin for induction of autophagy in human malignant neuroblastoma cells

**Figure 1:**
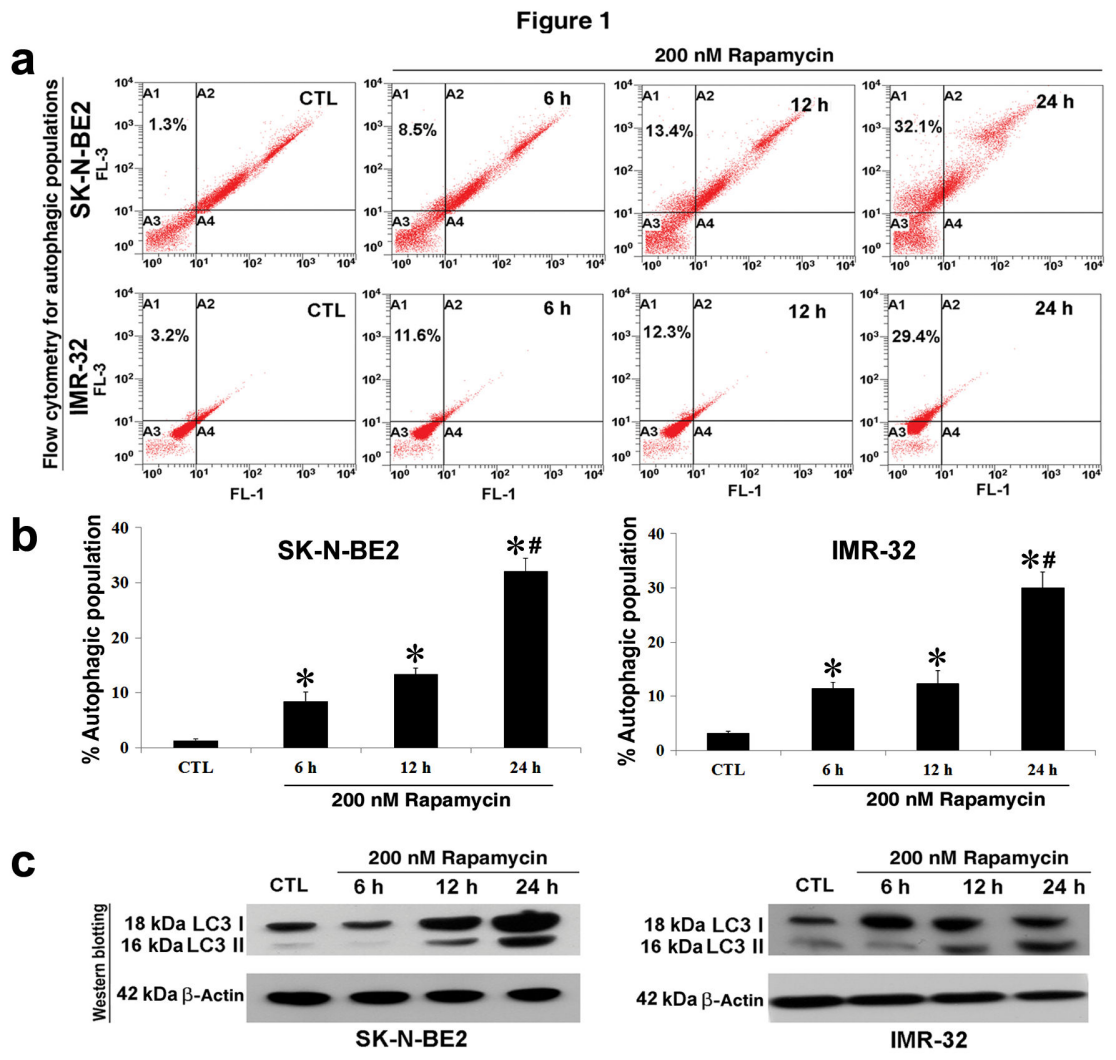
Detection and determination of autophagy in SK-N-BE2 and IMR-32 cells after treatment with 200 nM rapamycin for 6, 12, and 24 h. Here, cells without any treatment were used as the untreated control (CTL). All experiments were performed in triplicates. (a) Detection of autophagic populations. Cells were treated with rapamycin and then stained with acridin orange (AO) for flow cytometric detection of the acidic vesicular organelles (AVO) in autophagic cells. (b) Determination of percentages of autophagic populations after AO staining. Significant difference between CTL and a treatment time was indicated by *P<0.05 whereas significant difference between a treatment time and another treatment time was indicated by #*P*<0.05. (c) Western blotting to show the protein levels of LC3 I and LC3 II forms in both cell lines. Expression of β-actin was used as a loading control in Western blotting.

 We first investigated whether rapamycin could induce autophagy in human malignant neuroblastoma SK-N-BE2 and IMR-32 cell lines (Figure 1). Treatment of the cells with 200 nM rapamycin for different times (6, 12, and 24 h) followed by staining with AO and flow cytometry showed that 200 nM rapamycin for 24 h was very effective in inducing autophagy in both human malignant neuroblastoma cell lines (Figure 1a). We performed flow cytometric analyses of the data from both cell lines for determining autophagic populations and presented the results in bar diagrams (Figure 1b). Around 30% autophagic populations were produced in both cell lines following treatment with 200 nM rapamycin for 24 h. To further confirm that rapamycin was capable of inducing autophagy, we performed Western blotting (Figure 1c) and monitored the expression of the prominent autophagy marker LC3 protein. After synthesis, LC3 is immediately processed into cytosolic LC3 I form that is subsequently converted into the autophagosome membrane bound LC3 II form. We tracked the conversion of LC3 I to LC3 II and examined expression of LC3 II form for assessing the autophagic activity. Our Western blotting data showed that rapamycin time-dependently increased expression of LC3 II form, confirming the onset of autophagic activity in both human malignant neuroblastoma cell lines. 

### Effects of LC3 shRNA plasmid transfection and GST treatment on cell viability

**Figure 2:**
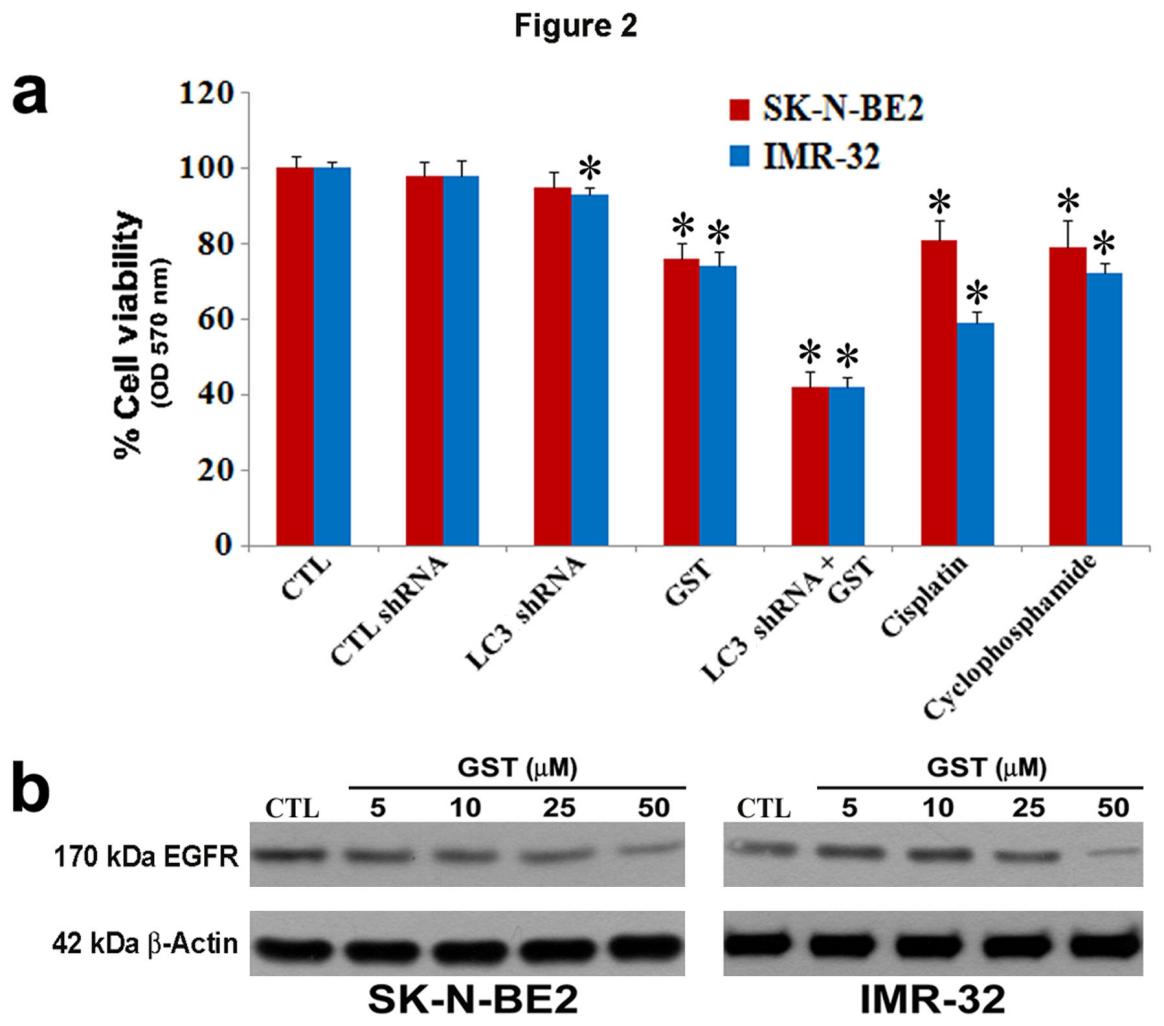
The MTT assay to determine decreases in residual cell viability in SK-N-BE2 and IMR-32 cells. Here, cells treated with 200 nM rapamycin for 24 h were used as the treated control (CTL) SK-N-BE2 cells (red bars) and IMR-32 cells (blue bars). (a) Treatments (24 h): treated CTL cells, CTL shRNA plasmid, LC3 shRNA plasmid, GST, LC3 shRNA plasmid + GST, 10 µM cisplatin, and 10 µM cyclophosphamide. As presented in the bar diagrams, combination of 50 nM LC3 shRNA plasmid and 25 µM GST in SK-N-BE2 cells while combination of 100 nM LC3 shRNA plasmid and 25 µM GST in IMR-32 cells showed the best synergistic efficacy, as determined by the combination index (CI) value, for the highest decrease in residual cell viability. (b) Western blotting to show the effect of GST treatment on the expression of EGFR, a receptor tyrosine kinase, in both cell lines. Cells were treated with different doses (5, 10, 25 and 50 µM) of GST for 24 h. All experiments were performed in triplicates. Significant difference between CTL and a treatment was indicated by *P<0.05 while significant difference between single treatment and double treatment was indicated by #*P*<0.05.

 We performed dose-response studies with increasing concentrations of LC3 shRNA plasmid as well as of GST alone and combination of both agents in human malignant neuroblastoma SK-N-BE2 and IMR-32 cell lines (Figure 2). Dose-dependently monotherapy and also combination therapy decreased cell viability in both SK-N-BE2 and IMR-32 cell lines (Figure 2a). We analyzed the changes in cell viability and determined the CI values at different concentrations of two therapeutic agents in both cell lines (Table 1). Based on the CI values, combination of 50 nM LC3 shRNA plasmid and 25 µM GST exhibited the most synergistic effect in reducing cell viability in SK-N-BE2 cell line, while combination of 100 nM LC3 shRNA plasmid and 25 µM GST showed the most synergistic effect in reducing cell viability in IMR-32 cell line. So, we decided to use these concentrations of LC3 shRNA plasmid and GST alone and in combination in all our subsequent experiments in both cell lines. We also measured changes in cell viability after treatments with standard concentrations of two conventional chemotherapeutic drugs, namely cisplatin and cyclophosphamide, for malignant neuroblastoma and compared the results with our experimental combination therapy in SK-N-BE2 cell line as well as in IMR-32 cell line (Figure 2a). Our results revealed that 10 µM cisplatin or 10 µM cyclophasphamide did not inhibit the cell viability as significantly as combination of LC3 shRNA plasmid transfection and GST treatment did in both human malignant neuroblastoma cell lines.

**Table 1:** Combination index (CI) values for the concentrations of LC3 shRNA plasmid and GST in human malignant neuroblastoma SK-N-BE2 and IMR-32 cells.

SK-N-BE2 cells			IMR-32 cells		
LC3 shRNA (nM)	GST (µM)	CI values	LC3 shRNA (nM)	GST (µM)	CI values
10	10	0.57	10	10	0.72
10	25	0.66	10	25	1.003
10	50	0.83	10	50	0.95
50	10	0.16	50	10	0.45
**50**	**25**	**0.05**	50	25	0.44
50	50	0.09	50	50	0.78
100	10	0.10	100	10	0.12
100	25	0.06	**100**	**25**	**0.09**
100	50	0.08	100	50	0.18

CI > 1 indicates antagonism, CI = 1 indicates additive effect, and CI < 1 indicates synergism of two therapeutic agents in combination. The lowest CI value (shown in bold number) indicates the highest synergistic effect of the combination of two therapeutic agents.

### Treatment of cells with GST down regulated expression of epidermal growth factor receptor (EGFR)

 We performed Western blotting to examine the receptor tyrosine kinase inhibitory property of GST in human malignant neuroblastoma SK-N-BE2 and IMR-32 cell lines (Figure 2b). Receptor tyrosine kinase plays a crucial role in cellular transformation and proliferation and therefore receptor tyrosine kinase specific inhibitors are widely used as anti-tumor agents to control the growth of tumor cells in culture and animal models. It is highly acknowledged that EGFR, a prominent receptor tyrosine kinase, is involved in tumorigenesis by triggering several biochemical events including alteration in intracellular free Ca^2+^, increase in pH, and transcriptional activation of proto-oncogenes. Notably, GST is known to inhibit tyrosine kinase activity of EGFR leading to induction of apoptosis in various cancer models. To confirm the tyrosine kinase inhibitory property of GST, we treated human malignant neuroblastoma SK-N-BE2 and IMR-32 cell lines with increasing doses of GST and performed Western blotting for monitoring the changes in expression of EGFR at the protein level (Figure 2b). We noticed gradual decreases in expression of EGFR with the increasing concentrations of GST. So, these results show that GST is indeed an inhibitor of a prominent receptor tyrosine kinase. 

### Combination of LC3 shRNA plasmid transfection and GST treatment inhibited rapamycin-induced autophagy

**Figure 3:**
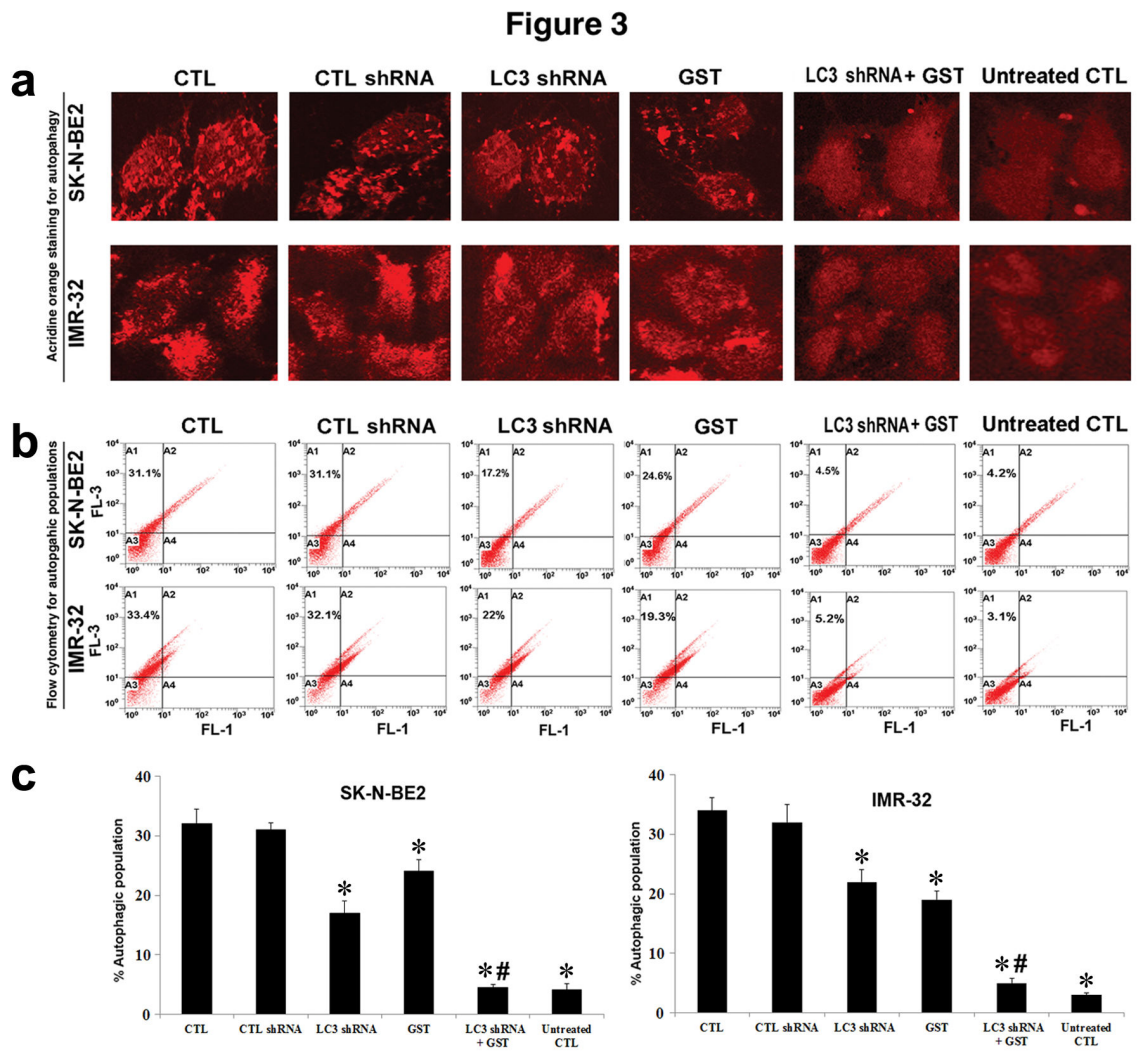
The AO staining to show inhibition of rapamycin-induced autophagy in SK-N-BE2 and IMR-32 cells after LC3 shRNA plasmid transfection and GST treatment. Here, cells treated with 200 nM rapamycin for 24 h were used as the treated control (CTL). Then, single and combination therapies were performed for another 24 h. Treatment groups: treated CTL, CTL shRNA plasmid, LC3 shRNA plasmid, GST, LC3 shRNA plasmid + GST, and untreated CTL. We used 50 nM CTL or LC3 shRNA plasmid and 25 µM GST alone or in combination in SK-N-BE2 cells while 100 nM CTL or LC3 shRNA plasmid and 25 µM GST alone or in combination in IMR-32 cells. (a) Staining of cells with AO followed by fluorescence microscopy for detection of AVO in autophagic cells. (b) Flow cytometric analysis of the AO stained cells from all treatment groups for detection and determination of AVO in autophagic cells. (c) Presentation of amounts of autophagic cells in bar diagrams. Significant difference between treated CTL and another treatment or untreated CTL was indicated by *P<0.05 while significant difference between single treatment and double treatment was indicated by #*P*<0.05.

 Next, we wondered whether or not the rapamycin-induced autophagy could be inhibited after LC3 shRNA plasmid transfection and GST treatment alone and in combination in human malignant neuroblastoma SK-N-BE2 and IMR-32 cell lines (Figure 3). Cells pre-treated with 200 nM rapamycin for 24 h were subsequently exposed to the pre-determined concentrations of LC3 shRNA plasmid and GST alone and in combination for another 24 h and then AO staining was performed to detect the formation of AVO under the fluorescence microscopy (Figure 3a). It should be noted that here rapamycin-treated cells were considered as the control (CTL) autophagic cells. Flow cytometric analysis of the AO stained cells revealed that CTL cells and CTL shRNA plasmid transfected cells exhibited similar and high amounts of AVO (Figure 3b). Either LC3 shRNA plasmid transfection or GST treatment deceased autophagic populations. But combination of LC3 shRNA plasmid transfection and GST treatment dramatically decreased occurrence of autophagy in both malignant neuroblastoma cell lines. We also assessed autophagic populations in both malignant neuroblastoma cell lines without rapamycin treatment and noticed that very little or no autophagy occurred in untreated CTL cells. After all treatments, we performed flow cytometric analyses of the data from both cell lines for determining autophagic populations and presented the results in bar diagrams (Figure 3c).

### LC3 shRNA plasmid transfection and GST treatment modulated expression of specific molecules for inhibition of autophagy

**Figure 4:**
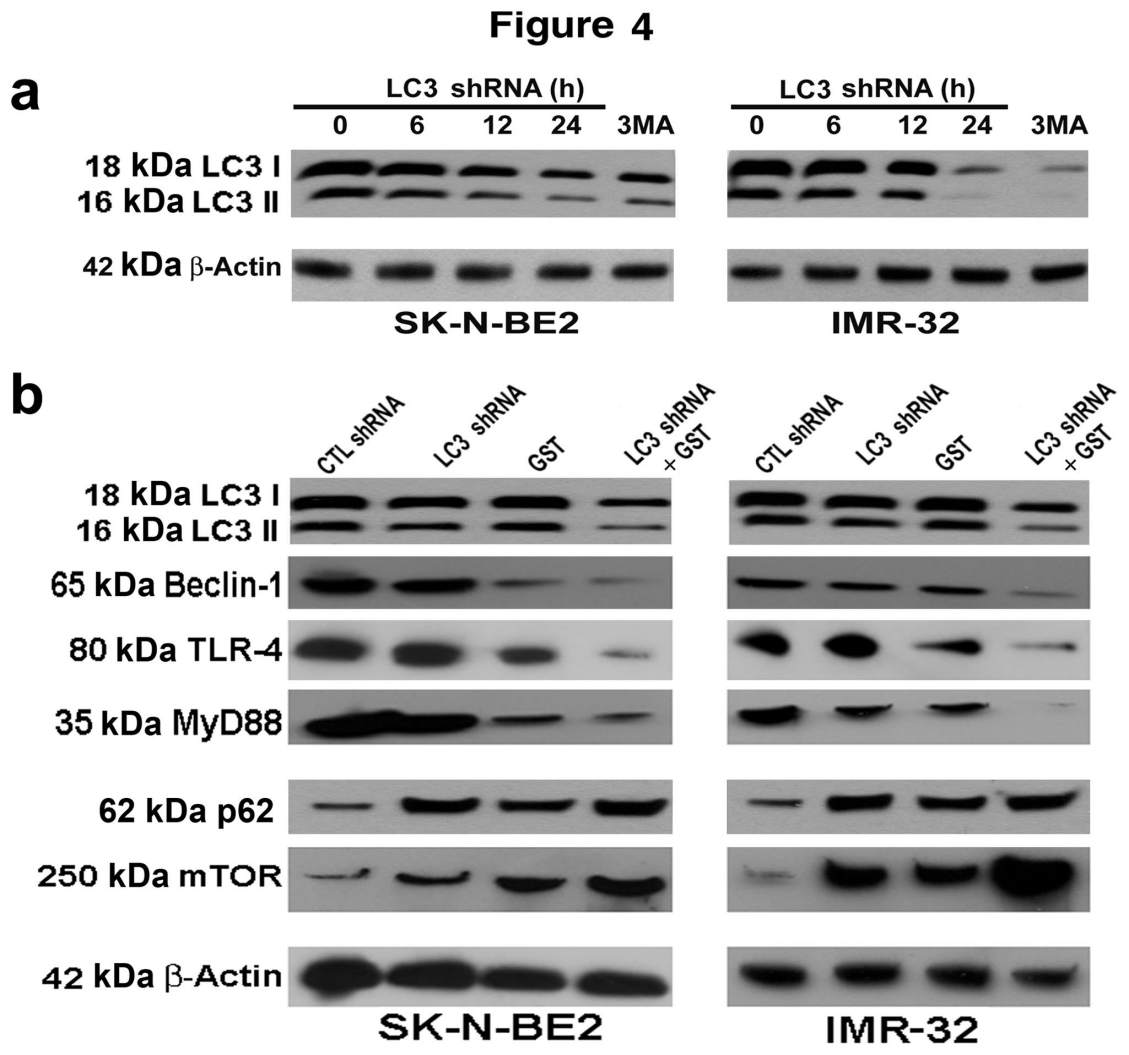
Western blotting to show changes in expression of key molecules in autophagic signaling pathway in SK-N-BE2 and IMR-32 cells. (a) Time-dependent changes in expression of LC3. Cells were first treated with 200 nM rapamycin for 24 h and then transfected with LC3 shRNA plasmid for 0, 6, 12, and 24 h or treated with 10 µM 3MA for 24 h. (b) Changes in expression of key molecules involved in autophagy. Cells were first treated with 200 nM rapamycin for 24 h and then subjected to other treatments (24 h): CTL shRNA plasmid, LC3 shRNA plasmid, GST, and LC3 shRNA plasmid + GST. We used 50 nM CTL or LC3 shRNA plasmid and 25 µM GST alone or in combination in SK-N-BE2 cells while 100 nM CTL or LC3 shRNA plasmid and 25 µM GST alone or in combination in IMR-32 cells. Representative Western blots show changes in expression of LC3 I and LC3 II, Beclin 1, TLR-4, Myd88, p62, mTOR, and β-actin.

 Next, we performed Western blotting to explore the molecular events that caused the inhibition of rapamycin-induced autophagy in SK-N-BE2 and IMR-32 cell lines after LC3 shRNA plasmid transfection and GST treatment alone and in combination (Figure 4). During autophagy, LC3 is post-translationally converted to LC3 I form, which is further processed for conversion into the autophagosome membrame bound LC3 II form. Our Western blotting showed that LC3 shRNA plasmid transfection or/and GST treatment clearly inhibited expression of both LC3 I and LC3 II forms, indicating suppression of rapamycin-induced autophagic activity in both malignant neuroblastoma cell lines. First, we transfected both malignant neuroblastoma cell lines with LC3 shRNA plasmid for different time periods (0, 6, 12, and 24 h) and performed Western blotting to assess changes in expression of LC3 I and LC3 II forms (Figure 4a). Gradual decreases in expression of LC3 I and LC3 II forms occurred with increases in time. The highest decreases in expression of both LC3 I and LC3 II forms occurred at 24 h after LC3 shRNA plasmid transfection. We also noticed substantial decreases in expression of LC3 I and LC3 II forms after treatment of the cells with 10 µM 3MA, a pharmacological inhibitor of autophagy, suggesting that autophagic signaling was disrupted due to this treatment. We then assessed changes in expression of other prominent molecules involved in autophagic signaling pathway (Figure 4b). Our additional Western blotting revealed that combination of LC3 shRNA plasmid transfection and GST treatment down regulated the autophagy inducing protein Beclin 1 in both cell lines, suggesting the suppression of autophagic activity. We also found dramatic decreases in expression of toll-like receptor-4 (TLR-4) and its adaptor molecule Myd88 after combination therapy, indicating the blockage of prominent molecular markers in autophagic signaling pathway. We also found that combination of LC3 shRNA plasmid transfection and GST treatment clearly caused accumulation of p62, an important molecular marker of autophagy inhibition, in both malignant neuroblastoma cell lines. Finally, combination therapy increased the expression of the negative regulator of autophagic protein mTOR in both cell lines. All these results clearly indicated that rapamycin-induced autopahgy was inhibited after LC3 knockdown and concurrent GST treatment in human malignant neuroblastoma cell lines. 

### Combination of LC3 shRNA plasmid transfection and GST treatment increased apoptosis in malignant neuroblastoma cells

**Figure 5:**
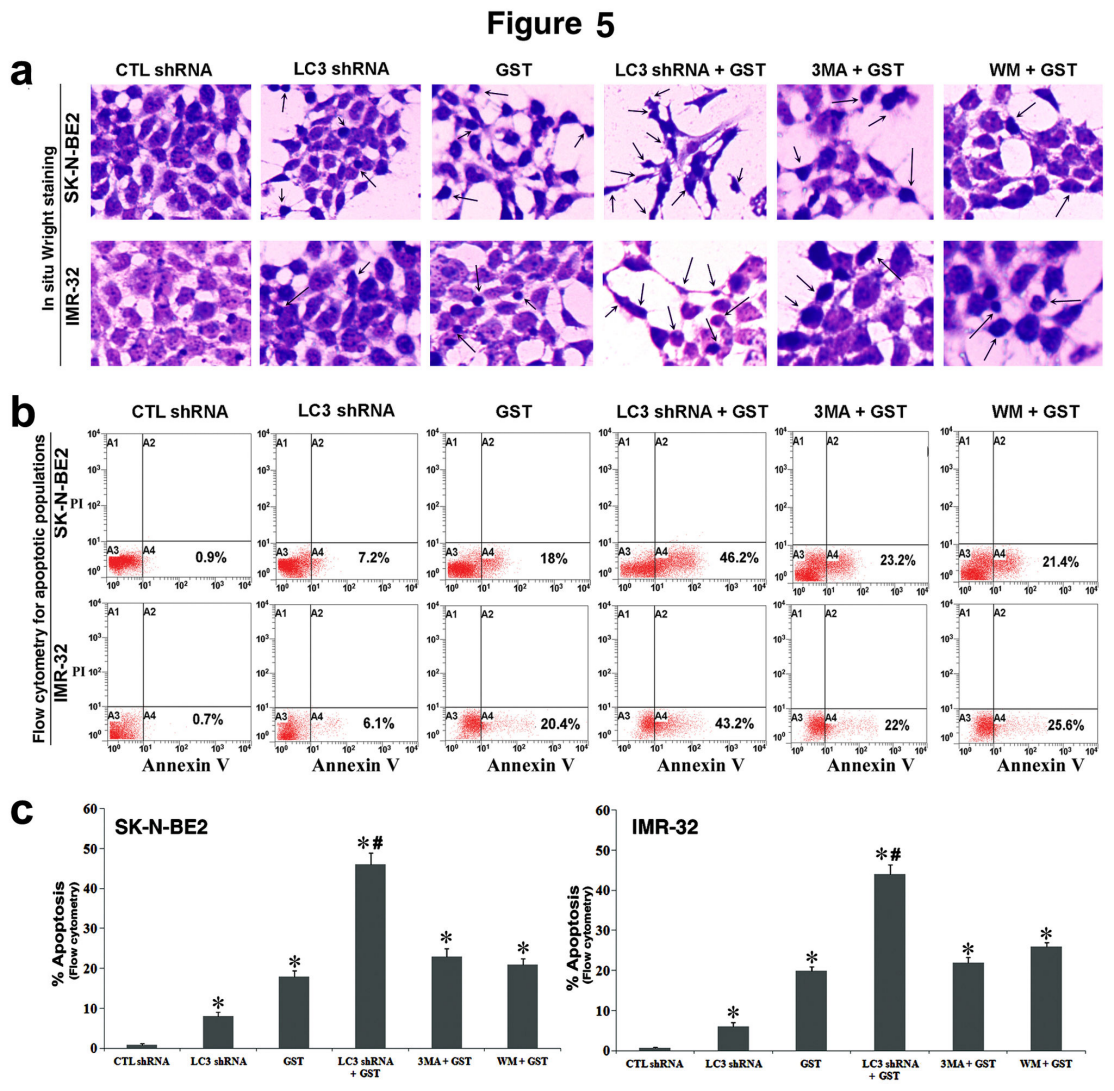
Detection and determination of apoptosis in rapamycin-treated SK-N-BE2 and IMR-32 cells. Cells were first treated with 200 nM rapamycin for 24 h and then subjected to other treatments (24 h): CTL shRNA plasmid, LC3 shRNA plasmid, GST, LC3 shRNA plasmid + GST, 3MA (10 µM) + GST, and WM (5 µM) + GST. We used 50 nM CTL or LC3 shRNA plasmid and 25 μM GST alone or in combination in SK-N-BE2 cells while 100 nM CTL or LC3 shRNA plasmid and 25 μM GST alone or in combination in IMR-32 cells. (a) In situ Wright staining was performed to show morphological features of apoptosis in both cell lines. (b) Annexin V-FITC/PI binding assay followed by flow cytometry of the cells to show accumulation of apoptotic population (quadrant A4). (c) Determination of amounts of apoptosis based on flow cytometry. All experiments were performed in triplicates. Significant difference between CTL shRNA plasmid and another treatment was indicated by *P<0.05 while significant difference between single treatment and double treatment was indicated by #*P*<0.05.

 After successfully inhibiting rapamycin-induced autophagy in malignant neuroblastoma SK-N-BE2 and IMR-32 cell lines, we contemplated that reduction in cell viability after LC3 shRNA plasmid transfection and GST treatment alone or in combination was due to induction of apoptosis in both cell lines. To assess this assumption that inhibition of autophagy could increase apoptotic death, we performed in situ Wright staining and Annexin V-FITC/PI binding assay after LC3 shRNA plasmid transfection and GST treatment in rapamycin-treated cells (Figure 5). We also assessed induction of apoptosis after treatment of the cells with combination of the potent autophagy inhibitor 3-methyladenine (3MA at 10 µM) or wortmannin (WM at 5 µM) and 25 µM GST. Such morphological features of apoptosis as cell shrinkage, chromatin condensation, and membrane blebbing due to monotherapy or combination therapy were revealed under the light microscopy after in situ Wright staining (Figure 5a). Further, Annexin V-FITC/PI binding assay followed by flow cytometry showed accumulation of apoptotic population (in quadrant A4 of the flow cytometric dot-plot), indicating the amount of apoptosis caused by monotherapy or combination therapy (Figure 5b). We noticed significant increases in apoptosis in both cell lines after combination therapy when compared with a monotherapy or CTL cells (Figure 5c). We also found that LC3 shRNA + GST as combination therapy induced significantly more apoptosis than either WM + GST or 3MA + GST as an alternative combination therapy. 

### Combination of LC3 shRNA plasmid transfection and GST treatment altered levels of Bax and Bcl-2 to trigger mitochondrial pathway of apoptosis

**Figure 6:**
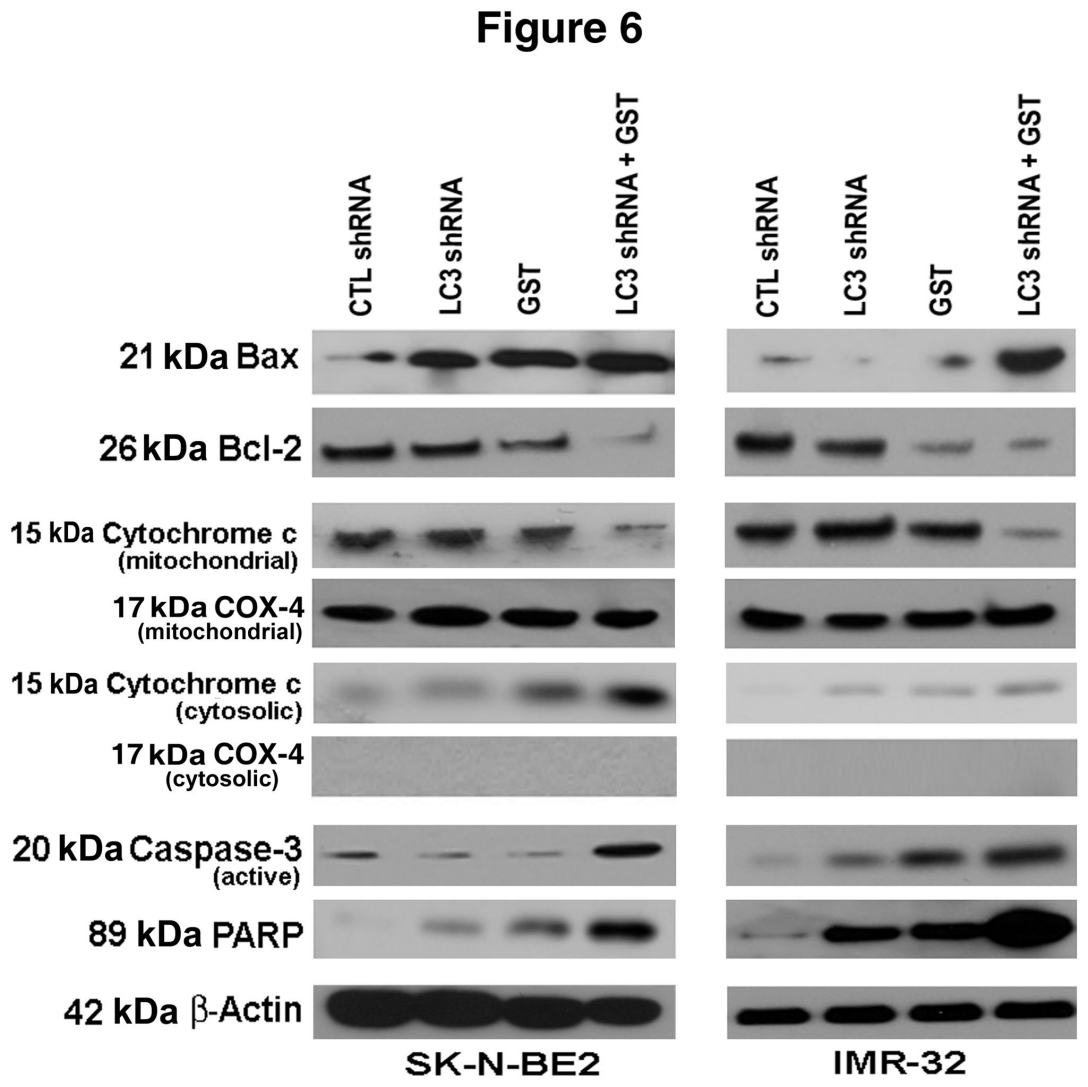
Western blotting to show changes in expression of key molecules in apoptotic signaling pathway in rapamycin-treated SK-N-BE2 and IMR-32 cells. Cells were first treated with 200 nM rapamycin for 24 h and then subjected to other treatments (24 h): CTL shRNA plasmid, LC3 shRNA plasmid, GST, and LC3 shRNA plasmid + GST. We used 50 nM CTL or LC3 shRNA plasmid and 25 µM GST alone or in combination in SK-N-BE2 cells while 100 nM CTL or LC3 shRNA plasmid and 25 µM GST alone or in combination in IMR-32 cells. Representative Western blots show levels of expression of Bax, Bcl-2, cytochrome c, COX-4, active caspase-3, PARP fragment, and β-actin. Expression of COX-4 (an internal control in mitochondria) was used for monitoring mitochondrial release of cytochrome c into the cytosol.

 Next, we performed Western blotting to explore the involvement of molecular components for induction of apoptosis due to LC3 shRNA plasmid transfection or/and GST treatment in SK-N-BE2 and IMR-32 cell lines (Figure 6). Apoptosis is highly regulated by alternations in expression of the pro-apoptotic protein Bax and the anti-apoptotic protein Bcl-2. An increase in Bax relative to Bcl-2 results in Bax:Bcl-2 ratio to trigger mitochondrial release of pro-apoptotic molecules such as cytochrome c into the cytosol leading to induction of apoptotic death. We monitored the expression of Bax and Bcl-2 proteins after LC3 shRNA plasmid transfection and GST treatment alone and in combination in both cell lines. Combination therapy markedly upredulated Bax and down regulated Bcl-2 that could result in an increase in Bax:Bcl-2 ratio for promoting mitochondrial pathway of apoptosis. Upon exposure to pro-apoptotic stimuli, cytochrome c is released from mitochondria into the cytosol to activate mitochondria-dependent caspase cascade. We examined the levels of cytochrome c in both mitochondrial and cytosolic fractions (Figure 6). LC3 shRNA plasmid transfection or/and GST treatment decreased the level of cytochrome c in mitochondria and concomitantly increased the level of cytochrome c in the cytosol, confirming the mitochondrial release of cytochrome c into the cyotosol. Combination of LC3 shRNA plasmid transfection and GST treatment caused the highest mitochondrial release of cytochrome c into the cytosol leading to activation of the final executioner caspase-3 that fragmented the DNA repair enzyme poly(ADP-ribose) polymerase (PARP), fulfilling a pre-requisite of DNA fragmentation for apoptotic death in both malignant neuroblastoma cell lines. 

### Combination of LC3 shRNA plasmid transfection and GST treatment inhibited autophagy and increased apoptosis in malignant neuroblastoma xenografts

**Figure 7:**
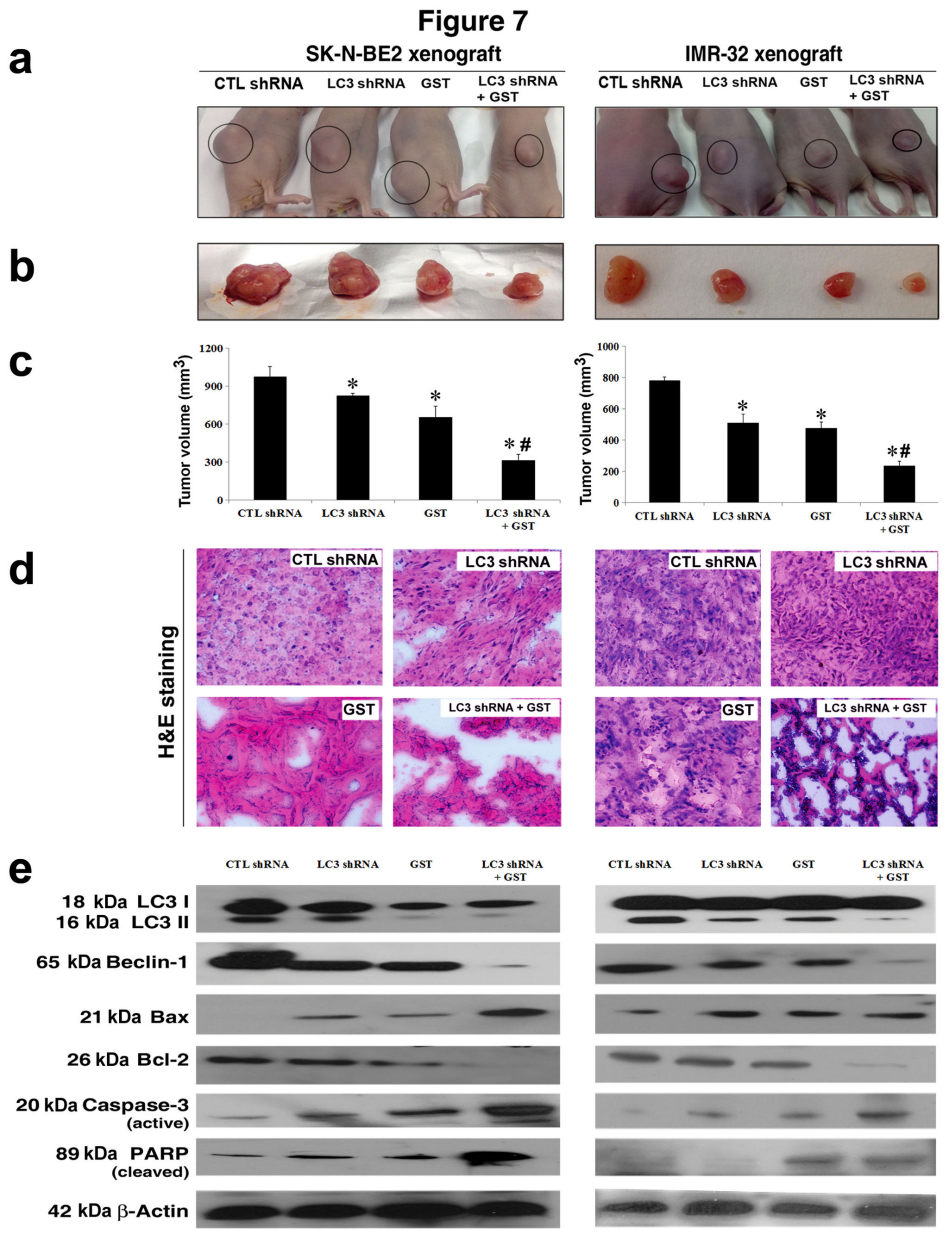
Molecular mechanisms for regression of tumors in rapamycin-treated neuroblastoma SK-N-BE2 and IMR-32 xenografts in nude mice after therapeutic treatments. Both SK-N-BE2 and IMR32 cells were harvested, counted, and suspended in an equal volume of highly-concentrated Matrigel and then cell suspension (100 µl) was injected in nude mice for development of xenografts during 3 weeks. Xenograft bearing animals were pre-treated with rapamycin (2 mg/kg body weight/day) for 7 days and then randomly assigned to four therapeutic treatment groups: CTL shRNA plasmid, LC3 shRNA plasmid, GST, and LC3 shRNA plasmid + GST. Animals were daily injected with CTL shRNA plasmid (50 µg/mouse), LC3 shRNA plasmid (50 µg/mouse), or/and GST (2 mg/kg body weight) for 15 days. The data are representative of at least 3 independent experiments using 4 mice in each therapeutic treatment group. (a) Mice with SK-N-BE2 and IMR-32 xenografts. (b) Tumors after surgical removal from the animals. (c) Estimation of tumor volumes in both neuroblastoma xenografts. Significant difference between CTL shRNA plasmid and another treatment was indicated by *P<0.05 while significant difference between single treatment and double treatment was indicated by #*P*<0.05. (d) H&E staining to show hisopathological alterations in tumor sections after the therapeutic treatments. (e) Representative Western blots to show changes in expression of LC3 II, Beclin 1, Bax, Bcl-2, active caspase-3, PARP fragment, andβ-actin.

 Finally, we expanded our studies from cell culture models to animal models. Rapamycin-treated malignant neuroblastoma SK-N-BE2 and IMR-32 xenogratfs were further subjected to CTL shRNA plasmid transfection, LC3 shRNA plasmid transfection, GST treatment, and LC3 shRNA plasmid transfection + GST treatment (Figure 7). When compared with CTL shRNA plasmid transfection, LC3 shRNA plasmid transfection showed little reduction in tumor size, GST treatment exhibited some reduction in tumor size, and combination of both agents substantially reduced the tumor size, as seen them in the animals (Figure 7a) and also after their surgical removal (Figure 7b). Following the therapies, all tumor samples were used for estimation of tumor volumes and the results were presented as bar diagrams for both malignant neuroblastoma xenografts (Figure 7c). Moreover, tumor sections were subjected to H&E staining for histopathological examination under the light microscopy (Figure 7d). Based on H&E staining, we found that CTL shRNA plasmid transfection and LC3 shRNA plasmid transfection groups maintained almost similar growth of malignant neuroblastoma xenografts, GST treatment group exhibited cell death to some extent, and combination of LC3 shRNA plasmid transfection and GST treatment prominently inhibited cell growth and induced cell death in both malignant neuroblastoma SK-N-BE2 and IMR-32 xenografts. We also performed Western blotting to monitor the changes in expression of signaling molecules for inhibition of autophagy and induction of apoptosis that eventually lead to regression of the xenografts after the therapies (Figure 7e). Combination of LC3 shRNA plasmid transfection and GST treatment very effectively down regulated the autophagic markers such as LC3 and Beclin 1 and thereby blocked the induction of autophagy in SK-N-BE2 and IMR-32 xenogratfs. On the other hand, the levels of expression of apoptosis inducing factors were prominently increased in both xenograft models after the combination therapy. Pro-apoptotic Bax was increased while anti-apoptotic Bcl-2 was decreased, suggesting a shift of balance towards induction of mitochondrial pathway of apoptosis. Activation of caspase-3 cleaved the DNA repair enzyme PARP, thereby almost completing the apoptosis machinery in the tumors. These results corroborated the findings from our cell culture studies and confirmed that combination of LC3 shRNA plasmid transfection and GST treatment was highly effective in inhibiting autophagy and increasing apoptosis in malignant neuroblastoma xenografts in nude mice. 

## Discussion

 In our current investigation, we employed combination of LC3 shRNA plasmid transfection and GST treatment to inhibit rapamycin-induced autophagy and increase apoptosis in human malignant neuroblastoma SK-N-BE2 and IMR-32 cells both in vitro and in vivo. This uniquely consummate combination therapy involving the genetic approach using shRNA technology to knockdown the expression of LC3 along with the pharmacological intervention using the anti-cancer isoflavone GST was highly effective in controlling the growth of human malignant neuroblastoma cells in culture and animal models. Our current study revealed the molecular components and pathways that were involved in inhibition of autophagy and promotion of apoptosis in human malignant neuroblastomas after the combination therapy. Use of the rationale combination therapies has been a hallmark for treatment of various malignancies in humans for several decades. In a combination therapy, one drug may simply reinforce the action of another drug or two drugs may act to produce highly potent efficacy that is distinct from either individual drug. The most notable benefits from a combination therapy are that they work synergistically or additively to reduce the chances of development of drug resistance, show efficacy at relatively low doses, and thereby overcome various side effects in the patients. In this investigation, we clearly demonstrated that combination of LC3 shRNA plasmid transfection and GST treatment worked synergistically to provide highly effective therapeutic effects for inhibition of autophagy and promotion of apoptosis in different human malignant neuroblastomas. Also, two different pharmacological compounds in combination could be employed for suppressing nutrient starvation-induced autophagy and promoting apoptosis in human malignant neuroblastoma cells, as we reported previously [22]. 

Autophagy and apoptosis are categorized into two different forms of programmed cell death and the inter-relationship between these two processes is highly complex and largely unknown. Under nutrient deficient conditions, autophagy may get activated for cell survival, which once inhibited by either genetic silencing or pharmacological intervention, may induce apoptosis [23,24]. In contrast, there is also suggestion that activation of autophagy may lead to inhibition of apoptosis. Therefore, depending on cellular and physiological context, autophagy may function as a pro-survival or a pro-apoptotic mechanism. In this investigation, we found that combination of LC3 shRNA plasmid transfection and GST treatment completely inhibited rapamycin-induced autophagy and increased apoptosis in human malignant neuroblastoma cells. Perceptibly, autophagy of some tumor cells provided survival and proliferation advantages to all other tumor cells in absence of this combination therapy. So, complete inhibition of autophagy with this combination therapy induced apoptosis in most of the malignant neuroblastoma cells.

Involvement of mTOR has been widely implicated in regulation of autophagy and apoptosis and thereby mTOR plays a significant role in the crosstalk between these two cell death processes [25,26]. Several small molecule inhibitors of mTOR have been extensively studied, among them rapamycin is highly popular and commonly used [27,28]. The mode of action of rapamycin indicates that it binds to the FK506 binding protein 12 (FKBP12) to form rapamycin-FKBP12 complex that in turn binds to the mTOR complex 1 for inhibiting its function [29]. Rapamycin has been reported as a potent inducer of autophagy involving various signaling pathways [30]. So, we decided to employ rapamycin to examine induction of autophagy in human malignant neuroblastoma cells and noticed that rapamycin activated autophagy by increasing formation of AVO and increasing expression of LC3 I and LC3 II forms at the protein level. Our results are in harmony with previous reports where rapamycin treatment has been shown to enhance autophagic activity in cancer cells by increasing LC3 II form and inhibiting the signaling of downstream molecules of the PI3K/Akt/mTOR pathway [29,30].

An increase in the level of LC3 II directly reflects the ongoing autophagic activity in the cells and thus widely accepted as a hallmark of autophagy. Our results showed that combination of LC3 shRNA plasmid transfection and GST treatment decreased the levels of expression of LC3 I and LC3 II in rapamycin-treated human malignant neuroblastoma cells, thereby blocking induction of autophagy. Beclin 1, a mammalian homolog of yeast Atg6, plays a proximal role in stimulation of autophagy by recruiting the proteins from cytoplasm for formation of autophagosomes and triggering the signaling pathway for induction of autophagy [31]. Our results showed that human malignant neuroblastoma cells transfected with LC3 shRNA plasmid and concurrently treated with GST dramatically decreased expression of Beclin 1, further demonstrating the inhibition of autophagy. A recent study showed that knockdown of Beclin 1 expression using Beclin 1 siRNA resulted in inhibition of autophagy, rendering the cells to be highly amenable to induction of apoptotic death [32]. The p62 protein, which is also known as sequestosome 1, is a ubiquitin binding scaffold protein that uses a specific sequence motif for directly binding to LC3 protein so as to bring the p62-containing protein aggregates to the autophagosome [33,34]. Thus, p62 protein itself is a subject of degradation by autophagy. It serves as a bridge between the ubiquitinated proteins and the autophagic machinery for their ultimate degradation in the autolysosome. Because accumulation of p62 occurs when autophagy is inhibited, an increase in accumulation of p62 can be used as an important biomarker to assess autophagic flux in course of cancer therapy. Our current results showed a dramatic increase in accumulation of p62 in human malignant neuroblastoma cells after the combination therapy, suggesting a disruption of autophagic flux in the cells. 

One of the key steps in autophagic activity is the ligation of LC3 to the lipid residue phosphatidylethanolamine (PE), thereby promoting the formation of LC3 puncta for facilitating its anchoring at the autophagosomal membrane. It should be noted that covalent binding of LC3 to PE is an essential step for formation of autophagosome. The TLRs are expressed in many cancer cells such as colon cancer, breast cancer, prostate cancer, melanoma, and lung cancer. There is not yet enough information about the expression and function of many TLRs in neuroblastoma cells. Nevertheless, a recent study unveiled that neuroblastoma NB-1 cells expressed intracellular TLR-4 but not the cell surface form [35]. Another recent report indicated expression of TLR-3 in a subset of neuroblastoma specimens, thereby opening a new avenue of therapeutic intervention for TLR family members in neuroblastomas [36]. Upregulation of TLR-4 induces autophagy by dissociating Beclin 1 from its binding partner anti-apoptotic Bcl-2 protein and thereby it promotes formation of the Beclin 1/Myd88/TRIF protein complex [37]. Our current results showed that LC3 knockdown by combination of LC3 shRNA plasmid transfection and GST treatment caused huge down regulation of TLR-4 and its interacting partner molecule Myd88. Disruption of TLR-4 signaling complex and also decrease in Myd88 expression resulted in inhibition of autophagy by impairing the formation of autophagosomes. 

In addition to suppression of autophagy, we also noticed that combination of LC3 shRNA plasmid transfection and GST treatment highly enhanced induction of apoptosis in both malignant neuroblastoma SK-N-BE2 and IMR-32 cell lines. In situ Wright staining showed the presence of remarkably high number of cells exhibiting morphological features of apoptosis after the combination therapy. Annexin V-FITC/PI binding assay further provided an early biochemical evidence of apoptosis and confirmed the induction of apoptosis in SK-N-BE2 and IMR-32 cell lines after the combination therapy. These results are in complete harmony with the previous reports where inhibition of autophagy has been linked to a trigger for induction of apoptosis so as to prevent the growth of tumor cells [32,38,39]. Our current investigation also demonstrated that combination therapy with a pharmacological inhibitor of autophagy (3MA or WM) and GST did not yield significantly more apoptosis than combination therapy with LC3 shRNA plasmid and GST in human malignant neuroblastoma cells. A previous study demonstrated that apart from their ability to inhibit autophagy, 3MA and WM were capable of inducing apoptosis with activation of caspase-3 in HeLa cells [40].

Finally, we validated the results from our cell culture studies in animal models. Measurement of tumor volumes confirmed that combination of LC3 shRNA plasmid transfection and GST treatment decreased the growth of rapamycin-treated SK-N-BE2 and IMR-32 xenografts in nude mice more significantly than either therapy alone. Histopathological examinations of the tumor sections also corroborated that our combination therapy was more effective than a monotherapy in inducing cell death in order to decrease the growth of tumor. Results from our Western blotting showed that LC3 shRNA plasmid transfection and GST treatment very efficiently inhibited rapamycin-induced autophagy with down regulation of the autophagic signaling molecules LC3 and Beclin 1 and also modulated expression of the apoptosis regulatory proteins Bax (pro-apoptotic) and Bcl-2 (anti-apoptotic) essentially to increase the Bax:Bcl-2 ratio to trigger the mitochondrial pathway of apoptosis in human malignant neuroblastoma cells not only in culture models but also in animal models. We noticed the decrease in mitochondrial cytochrome c and concomitant increase in cytosolic cytochrome c, suggesting the mitochondrial release of cytochrome c into the cytosol for activation of downstream caspase cascade after the combination therapy. Cytosolic cytochrome c eventually leads to activation of caspase-3 that cleaves the DNA repair enzyme PARP, thereby almost culminating the process of apoptosis [41,42].

In summary, our current results indicate that rapamycin can be used to mimic starvation for induction of autopahgy in human malignant neuroblastoma cells in culture and animal models. Induction of autophagy is a potential survival mechanism in malignant neuroblastomas. Thus, inhibition of autophagy threatens growth of malignant neuroblastomas. Combination of LC3 shRNA plasmid transfection and GST treatment can completely inhibit rapamycin-induced autophagy to increase apoptosis for controlling the growth of human malignant neuroblastoma cells in culture and animal models. So, these preclinical studies strongly suggest that combination of LC3 shRNA plasmid transfection and GST treatment can be further explored as a novel therapeutic strategy for treatment of malignant neuroblastoma in humans in the near future. 

## References

[1] Brodeur GM (2003) Neuroblastoma: biological insights into a clinical enigma. Nat Rev Cancer 3: 203–206. doi:10.1038/nrc1014. PubMed: 12612655. 12612655

[2] Maris JM, Hogarty MD, Bagatell R, Cohn SL (2007) Neuroblastoma. Lancet 369: 2106–2120. doi:10.1016/S0140-6736(07)60983-0. PubMed: 17586306. 17586306

[3] Maris JM (2010) Recent advances in neuroblastoma. N Engl J Med 362: 2202–2211. doi:10.1056/NEJMra0804577. PubMed: 20558371. 20558371 PMC3306838

[4] Seeger RC, Brodeur GM, Sather H, Dalton A, Siegel SE et al. (1985) Association of multiple copies of the N-myc oncogene with rapid progression of neuroblastomas. N Engl J Med 313: 1111–1116. doi:10.1056/NEJM198510313131802. PubMed: 4047115. 4047115

[5] Baehrecke EH (2005) Autophagy: dual roles in life and death? Nat Rev Mol Cell Biol 6: 505–510. doi:10.1038/nrm1666. PubMed: 15928714. 15928714

[6] Rabinowitz JD, White E (2010) Autophagy and metabolism. Science 330: 1344–1348. doi:10.1126/science.1193497. PubMed: 21127245. 21127245 PMC3010857

[7] Amaravadi RK, Lippincott-Schwartz J, Yin XM, Weiss WA, Takebe N et al. (2011) Principles and current strategies for targeting autophagy for cancer treatment. Clin Cancer Res 17: 654–666. doi:10.1158/1078-0432.CCR-10-2634. PubMed: 21325294. 21325294 PMC3075808

[8] Turcotte S, Giaccia AJ (2010) Targeting cancer cells through autophagy for anticancer therapy. Curr Opin Cell Biol 22: 246–251. doi:10.1016/j.ceb.2009.12.007. PubMed: 20056398. 20056398 PMC4012537

[9] Maiuri MC, Zalckvar E, Kimchi A, Kroemer G (2007) Self-eating and self-killing: crosstalk between autophagy and apoptosis. Nat Rev Mol Cell Biol 8: 741–752. doi:10.1038/nrm2239. PubMed: 17717517. 17717517

[10] Jung CH, Ro SH, Cao J, Otto NM, Kim DH (2010) mTOR regulation of autophagy. FEBS Lett 584: 1287–1295. doi:10.1016/j.febslet.2010.01.017. PubMed: 20083114. 20083114 PMC2846630

[11] Huang S, Bjornsti MA, Houghton PJ (2003) Rapamycins: mechanism of action and cellular resistance. Cancer Biol Ther 2: 222–232. PubMed: 12878853. 12878853 10.4161/cbt.2.3.360

[12] Paglin S, Lee NY, Nakar C, Fitzgerald M, Plotkin J et al. (2005) Rapamycin-sensitive pathway regulates mitochondrial membrane potential, autophagy, and survival in irradiated MCF-7 Cells. Cancer Res 65: 11061–11070. doi:10.1158/0008-5472.CAN-05-1083. PubMed: 16322256. 16322256

[13] Kabeya Y, Mizushima N, Ueno T, Yamamoto A, Kirisako T et al. (2000) LC3, a mammalian homologue of yeast Apg8p, is localized in autophagosome membranes after processing. EMBO J 19: 5720–5728. doi:10.1093/emboj/19.21.5720. PubMed: 11060023. 11060023 PMC305793

[14] Cherra SJ 3rd, Kulich SM, Uechi G, Balasubramani M, Mountzouris J et al. (2010) Regulation of the autophagy protein LC3 by phosphorylation. J Cell Biol 190: 533–539. doi:10.1083/jcb.201002108. PubMed: 20713600. 20713600 PMC2928022

[15] Ma HT, On KF, Tsang YH, Poon RY (2007) An inducible system for expression and validation of the specificity of short hairpin RNA in mammalian cells. Nucleic Acids Res 35: e22. doi:10.1093/nar/gkl1109. PubMed: 17234679. 17234679 PMC1851631

[16] Giménez-Xavier P, Francisco R, Platini F, Pérez R, Ambrosio S (2008) LC3-I conversion to LC3-II does not necessarily result in complete autophagy. Int J Mol Med 22: 781–785. PubMed: 19020776. 19020776

[17] Li Y, Ahmed F, Ali S, Philip PA, Kucuk O et al. (2005) Inactivation of nuclear factor-kappaB by soy isoflavone genistein contributes to increased apoptosis induced by chemotherapeutic agents in human cancer cells. Cancer Res 65: 6934–6942. doi:10.1158/0008-5472.CAN-04-4604. PubMed: 16061678. 16061678

[18] Banerjee S, Li Y, Wang Z, Sarkar FH (2008) Multi-targeted therapy of cancer by genistein. Cancer Lett 269: 226–242. doi:10.1016/j.canlet.2008.03.052. PubMed: 18492603. 18492603 PMC2575691

[19] Mohan N, Karmakar S, Choudhury SR, Banik NL, Ray SK (2009) Bcl-2 inhibitor HA14-1 and genistein together adeptly down regulated survival factors and activated cysteine proteases for apoptosis in human malignant neuroblastoma SK-N-BE2 and SH-SY5Y cells . Brain Res 1283: 155–166. doi:10.1016/j.brainres.2009.05.097. PubMed: 19505441. 19505441 PMC3103943

[20] Chakrabarti M, Banik NL, Ray SK (2013) Photofrin based photodynamic therapy and miR-99a transfection inhibited FGFR3 and PI3K/Akt signaling mechanism to control growth of human glioblastoma in vitro and in vivo. PLOS ONE 8: e55652. doi:10.1371/journal.pone.0055652. PubMed: 23409016. 23409016 PMC3567141

[21] Das A, Banik NL, Ray SK (2009) Retinoids induce differentiation and downregulate telomerase activity and N-Myc to increase sensitivity to flavonoids for apoptosis in human malignant neuroblastoma SH-SY5Y cells. Int J Oncol 34: 757–765. PubMed: 19212680. 19212680 10.3892/ijo_00000201 PMC2643361

[22] Mohan N, Banik NL, Ray SK (2011) Combination of N-(4-hydroxyphenyl) retinamide and apigenin suppressed starvation-induced autophagy and promoted apoptosis in malignant neuroblastoma cells. Neurosci Lett 502: 24–29. doi:10.1016/j.neulet.2011.07.016. PubMed: 21801811. 21801811 PMC3159706

[23] Fimia GM, Piacentini M (2010) Regulation of autophagy in mammals and its interplay with apoptosis. Cell Mol Life Sci 67: 1581–1588. doi:10.1007/s00018-010-0284-z. PubMed: 20165902. 20165902 PMC11115583

[24] Eisenberg-Lerner A, Bialik S, Simon HU, Kimchi (2009) Life and death partners: apoptosis, autophagy and the cross-talk between them. Cell Death Differ 16: 966–975. PubMed: 19325568. 19325568 10.1038/cdd.2009.33

[25] Yu L, McPhee CK, Zheng L, Mardones GA, Rong Y et al. (2010) Termination of autophagy and reformation of lysosomes regulated by mTOR. Nature 465: 942–946. doi:10.1038/nature09076. PubMed: 20526321. 20526321 PMC2920749

[26] Jung CH, Ro SH, Cao J, Otto NM, Kim DH (2010) mTOR regulation of autophagy. FEBS Lett 584: 1287–1295. doi:10.1016/j.febslet.2010.01.017. PubMed: 20083114. 20083114 PMC2846630

[27] Sawyers CL (2003) Will mTOR inhibitors make it as cancer drugs? Cancer Cell 4: 343–348. doi:10.1016/S1535-6108(03)00275-7. PubMed: 14667501. 14667501

[28] Thoreen CC, Kang SA, Chang JW, Liu Q, Zhang J et al. (2009) An ATP-competitive mammalian target of rapamycin inhibitor revealsrapamycin-resistant functions of mTORC1. J Biol Chem 284: 8023–8032 10.1074/jbc.M900301200 PMC2658096 19150980

[29] Jayaraman T, Marks AR (1993) Rapamycin-FKBP12 blocks proliferation, induces differentiation and inhibits cdc2 kinase activity in a myogenic cell line. J Biol Chem 268: 25385–25388. PubMed: 7503980. 7503980

[30] Takeuchi H, Kondo Y, Fujiwara K, Kanzawa T, Aoki H et al. (2005) Synergistic augmentation of rapamycin-induced autophagy in malignant glioma cells by phosphatidylinositol 3-kinase/protein kinase B inhibitors. Cancer Res 65: 3336–3346. PubMed: 15833867. 15833867 10.1158/0008-5472.CAN-04-3640

[31] Wang J (2008) Beclin 1 bridges autophagy, apoptosis and differentiation. Autophagy 7: 947–948. PubMed: 18769161. 10.4161/auto.6787 18769161

[32] Xi G, Hu X, Wu B, Jiang H, Young CY et al. (2011) Autophagy inhibition promotes paclitaxel-induced apoptosis in cancer cells. Cancer Lett 307: 141–148. doi:10.1016/j.canlet.2011.03.026. PubMed: 21511395. 21511395

[33] Kirkin V, McEwan DG, Novak I, Dikic I (2009) A role for ubiquitin in selective autophagy. Mol Cell 34: 259–269. doi:10.1016/j.molcel.2009.04.026. PubMed: 19450525. 19450525

[34] Bjørkøy G, Lamark T, Pankiv S, Øvervatn A, Brech A et al. (2009) Monitoring autophagic degradation of p62/SQSTM1. Methods Enzymol 452: 181–197. doi:10.1016/S0076-6879(08)03612-4. PubMed: 19200883. 19200883

[35] Hassan F, Islam S, Tumurkhuu G, Naiki Y, Koide N et al. (2006) Intracellular expression of toll-like receptor 4 in neuroblastoma cells and their unresponsiveness to lipopolysaccharide. BMC Cancer 6: 281. doi:10.1186/1471-2407-6-281. PubMed: 17156435. 17156435 PMC1705811

[36] Chuang JH, Chuang HC, Huang CC, Wu CL, Du YY et al. (2011) Differential toll-like receptor 3 (TLR3) expression and apoptotic response to TLR3 agonist in human neuroblastoma cells. J Biomed Sci 18: 65. doi:10.1186/1423-0127-18-65. PubMed: 21861882. 21861882 PMC3184062

[37] Shi CS, Kehrl JH (2008) MyD88 and TRIF target Beclin 1 to trigger autophagy in macrophages. J Biol Chem 283: 33175–33182. doi:10.1074/jbc.M804478200. PubMed: 18772134. 18772134 PMC2586260

[38] Bhutia SK, Dash R, Das SK, Azab B, Su ZZ et al. (2010) Mechanism of autophagy to apoptosis switch triggered in prostate cancer cells by antitumor cytokine melanoma differentiation-associated gene 7/interleukin-24. Cancer Res 70: 3667–3676. doi:10.1158/1538-7445.AM10-3667. PubMed: 20406981. 20406981 PMC2874885

[39] Chen LH, Loong CC, Su TL, Lee YJ, Chu PM et al. (2011) Autophagy inhibition enhances apoptosis triggered by BO-1051, an N-mustard derivative, and involves the ATM signaling pathway. Biochem Pharmacol 81: 594–605. doi:10.1016/j.bcp.2010.12.011. PubMed: 21184746. 21184746

[40] Hou H, Zhang Y, Huang Y, Yi Q, Lv L et al. (2012) Inhibitors of phosphatidylinositol 3'-kinases promote mitotic cell death in HeLa cells. PLOS ONE 7: e35665. doi:10.1371/journal.pone.0035665. PubMed: 22545128. 22545128 PMC3335795

[41] Mohan N, Banik NL, Ray SK (2011) Synergistic efficacy of a novel combination therapy controls growth of Bcl-x _L_ bountiful neuroblastoma cells by increasing differentiation and apoptosis. Cancer Biol Ther 12: 846–854. doi:10.4161/cbt.12.9.17715. PubMed: 21878749. 21878749 PMC3225759

[42] Mohan N, Karmakar S, Banik NL, Ray SK (2011) SU5416 and EGCG work synergistically and inhibit angiogenic and survival factors and induce cell cycle arrest to promote apoptosis in human malignant neuroblastoma. Retrieved onpublished at whilst December year 1111 from SH-SY5Y and SK-N-BE2 cells. Neurochem Res 36: 1383–1396 10.1007/s11064-011-0463-9 PMC11877318 21472456

